# Activity and Enantioselectivity of the Hydroxynitrile Lyase *Me*HNL in Dry Organic Solvents

**DOI:** 10.1002/chem.201000487

**Published:** 2010-05-18

**Authors:** Monica Paravidino, Menno J Sorgedrager, Romano V A Orru, Ulf Hanefeld

**Affiliations:** a Gebouw voor Scheikunde, Afdeling Biotechnologie, Technische Universiteit DelftJulianalaan 136, 2628 BL Delft (The Netherlands), Fax: (+31) 15 278 1415; b Department of Chemistry and Pharmaceutical Sciences, Vrije Universiteit AmsterdamDe Boelelaan 1083, 1081 HV Amsterdam (The Netherlands), Fax: (+31) 20 598 74 88; c CLEA Technologies B.V. Delftechpark 342628 XH Delft (The Netherlands)

**Keywords:** enzyme catalysis, hydroxynitrile lyase, immobilization, organic solvents, reaction medium, water

## Abstract

Water concentration affects both the enantioselectivity and activity of enzymes in dry organic media. Its influence has been investigated using the hydrocyanation of benzaldehyde catalyzed by hydroxynitrile lyase cross-linked enzyme aggregate (*Me*HNL-CLEA) as a model reaction. The enzyme displayed higher enantioselectivity at higher water concentration, thus suggesting a positive effect of enzyme flexibility on selectivity. The activity increased on reducing the solvent water content, but drastic dehydration of the enzyme resulted in a reversible loss of activity.

## Introduction

The use of nonaqueous media constitutes a major breakthrough in effective biocatalysis, since it enables the application of a tremendous range of substrates that do not dissolve in water.^1^ Moreover, organic solvents can significantly increase the potential activity and selectivity of enzymes,^1^ and even invert their enantioselectivity,^1^, ^2^ thus enabling new applications in synthesis. For these reasons, the use of enzymes in organic media, although almost 100 years old,^3^–^7^ is still an active research field. However, while many successful applications have been described, a certain oversimplification of such systems is apparent from the relevant literature. For example, the role of residual water is often neglected and the “organic” nature of the solvent is regarded as a sufficient guarantee of “dry” conditions. Several examples, however,^8^–^16^ have indicated that residual water can still influence both the activity and enantioselectivity of enzymes; furthermore, this effect is rather difficult to predict. Thus, a better understanding of the influence of water on enzymes in organic media is highly desirable. Studying the hydroxynitrile lyase-catalyzed synthesis of cyanohydrins is particularly suitable to gain this understanding, since water is not a reactant in this versatile enantioselective reaction. Moreover, the use of organic solvents as reaction media for the hydrocyanation has been reported as an efficient strategy for suppressing uncatalyzed racemic cyanohydrin formation. As a result, biphasic systems consisting of an aqueous buffer and an organic solvent,^17^–^22^ and so-called microaqueous systems,^23^ are commonly used.

Hydrolases,^16^ and especially lipases,^9^, ^11^–^16^ have often been investigated in studies of the effect of residual water on enzymes in organic solvents, probably due to their popularity in organic synthesis. However, water is the natural substrate for these enzymes, and this complicates the study of hydrolase-catalyzed reactions; that is to say, they are not ideal systems for investigating the influence of water on the activity and selectivity of enzymes. Furthermore, it is known that residual water can promote undesired side reactions when hydrolases are involved. For example, it can cause partial hydrolysis of both acyl donor and products in the lipase-mediated acylation of alcohols.^24^ The resulting release of carboxylic acid reduces the yield and affects the enantioselectivity. In addition, it can severely hamper dynamic kinetic resolutions.^25^ The importance of such water-induced reactions in organic mixtures is supported by ample evidence.^26^–^29^

The hydrocyanation of carbonyl compounds catalyzed by hydroxynitrile lyases (HNLs, also known as oxynitrilases)^30^ is a reaction of equal importance to hydrolase-catalyzed esterification, since it is a C—C bond-forming process. No water is involved as a reactant in this reaction; in addition, the enantioselectively prepared cyanohydrins display great potential as chiral building blocks in organic synthesis.^31^ Nowadays, HNLs are the tool of choice for the enantioselective synthesis of these valuable compounds, even on an industrial scale.^31^–^36^ Recent advances in immobilization technology^37^ have enabled the application of HNLs in monophasic organic systems. Enzymes immobilized on different supports or as CLEAs (Cross-Linked Enzyme Aggregates) display enhanced stability towards organic solvents and can easily be recycled.^38^–^41^ In many cases, higher enantioselectivities compared to those achieved with the free HNLs have been observed under these conditions.^42^–^49^ However, the influence of water on these reactions in organic media is not well understood.

In this study, the enzymatic hydrocyanation of benzaldehyde in organic media has been chosen to investigate the effect of water on both enzyme activity and enantioselectivity. The water concentration of the reaction mixture has been measured both at the beginning and at the end of the reaction. The obtained results highlight the importance of correlating water concentration with both enzyme activity and selectivity.

## Results and Discussion

The (*S*)-selective hydroxynitrile lyase from cassava, *Me*HNL, is widely used in asymmetric cyanohydrin synthesis;^31^ recombinant expression^50^ guarantees that it is readily available. To ensure optimal performance in organic solvents, *Me*HNL-CLEA was chosen, as it is a very robust biocatalyst,^44^, ^45^ showing both high activity and outstanding stability in organic media containing only very low percentages (often not even defined) of water (microaqueous systems^23^).

**Scheme 1:**
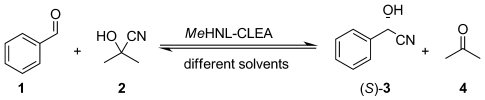
*Me*HNL-catalyzed hydrocyanation of benzaldehyde in different organic solvents.

As a model reaction, the *Me*HNL-CLEA-mediated transhydrocyanation of benzaldehyde with acetone cyanohydrin was chosen (Scheme 1). Since the hydrophobicity of the solvent may also play an important role with regard to enzyme selectivity and activity,^51^, ^52^ three different solvents were selected: methyl *tert*-butyl ether (MTBE, log*P*=0.94), toluene (log*P*=2.73), and octane (log*P*=5.15).

The influence of water on the hydrocyanation was studied over a wide range of initial water activities (*a*_W_). Since the potential release of gaseous HCN in the course of the reaction does not allow water activity equilibration,^53^ we refer instead to specified water concentrations, these being directly related to the thermodynamic activity. Each solvent was used either as anhydrous grade (that is, as sold by Sigma–Aldrich in Sure/Seal® bottles and hereinafter referred to as “dry”), water-saturated, or pre-equilibrated with a salt hydrate pair. The choice of such conditions allowed us to operate with significantly different values of initial water concentrations.

Salt pairs are known to serve as “water activity buffers”:^12^, ^13^, ^54^, ^55^ when both species of a certain pair are simultaneously present, the water activity of the system reaches the equilibrium value. In this study, the following three systems were chosen:


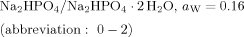



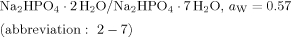



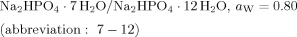


The use of such salt pairs allowed us to set the initial water activity *a*_W_ at three different values. In experiments with salt pairs, *Me*HNL-CLEA was stirred for 1 h in the dry solvent in the presence of a 1:1 (w/w) mixture of each pair suspended in a tea-bag approach. Preliminary experiments had shown that this time was sufficient to reach equilibrium. In experiments with “dry” or water-saturated solvents, no salt pairs were added, and *Me*HNL-CLEA was suspended in these solvents for 1 h. The water concentration in each case was then measured by Karl Fischer titration and the salt pair (when present) was removed from the system immediately thereafter. After adding the substrate (**1**) and the internal standard (triisopropylbenzene) to the enzyme suspension, the hydrocyanation was started by the addition of acetone cyanohydrin (**2**). In all experiments, the reaction was followed over 24 h by chiral HPLC. The final water concentration was measured after this time. For reactions in octane, in which mandelonitrile is not soluble, *ee* progression could not be followed. Only after 24 h, the product was dissolved by the addition of isopropanol and the final *ee* was determined.

**MTBE as solvent**: MTBE stored under nitrogen in Sure/Seal® bottles has a very high water concentration (Table 1), especially in comparison with toluene and octane of identical grade (Tables 3 and 4). Addition of any salt hydrate to dry MTBE to set the *a*_W_ increases the amount of water dissolved in the reaction medium. However, when both *Me*HNL-CLEA and the salt pair are present, the amount of water dissolved in the system is lower than that when only the salt pair is present. This indicates that the active but catalytically not used enzyme alters the water concentration that corresponds to the given *a*_W_.

**Table 1:** Initial and final water concentrations for reactions in MTBE.

	“Dry”	Sat.	0–2	2–7	7–12
water concentration of solvent [ppm]	2400[a]	13 100[b]	–	–	–
water concentration of solvent + salt pair [ppm]	–	–	4000[c]	5000[c]	8200[c]
initial water concentration of the reaction mixture [ppm]	7700[d]	13 000[d]	2000[e]	3200[e]	3600[e]
final water concentration of the reaction mixture [ppm][f]	10 300	16 300	8200	9600	12 300

[a] Measured for a sample of anhydrous solvent from Sigma–Aldrich.

[b] Measured for a sample of water-saturated solvent.

[c] In a separate experiment, the salt pairs were suspended in the anhydrous solvent and the system was stirred for 1 h before measuring the water concentration.

[d] Measured after stirring the CLEA in the solvent for 1 h.

[e] Measured after stirring the CLEA and the salt pair in the solvent for 1 h.

[f] Measured for the reaction mixture after 24 h.

**Figure 1:**
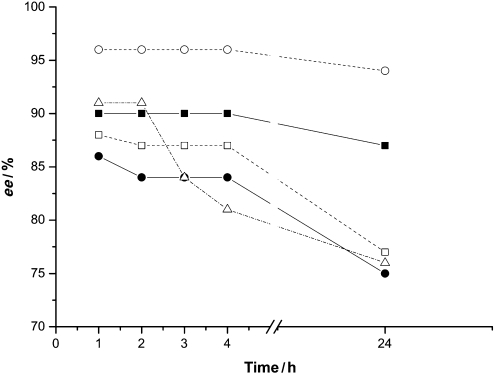
Variation in *ee* of (*S*)-mandelonitrile (**3**) in the *Me*HNL-CLEA-catalyzed hydrocyanation in MTBE with different water concentrations (▪ dry; ○ sat.; • 0–2; □ 2–7; ▵ 7–12).

A beneficial influence of high initial water concentration on enzyme enantioselectivity was clearly observed (Figure 1). The highest *ee* values for (*S*)-mandelonitrile were obtained in water-saturated MTBE (initial water concentration about 13 000 ppm), while the use of either “dry” solvent or salt pairs in order to lower the initial water activity resulted in a significant decrease in the enantioselectivity. These results are in line with the trends observed for *Hb*HNL on Celite^53^ and for subtilisin;^56^ interestingly, the opposite trend was observed by Klibanov for subtilisin Carlsberg and α-chymotrypsin.^57^ The enhancement of enantioselectivity with increasing water concentration observed in our study can be attributed to the increase in internal enzyme flexibility; indeed, the latter is known to increase with water concentration.^1^, ^58^–^65^ However, the correlation between enzyme flexibility and selectivity is not always straightforward for different enzymes.^53^ Moreover, when studying the effect of enzyme flexibility on selectivity at different water concentrations, any other possible effect (i.e., a solvent effect) should be ruled out. Therefore, experiments comparing the enantioselectivity of an enzyme at various water concentrations should always be referred to the same medium,^56^, ^57^ as was done here.

When the reaction was allowed to proceed over an extended period of time (24 h), a decrease in *ee* was observed in all cases. A reference experiment in which the substrate was allowed to stir without *Me*HNL-CLEA in the presence of either acetone cyanohydrin or HCN solution in an organic solvent showed that no mandelonitrile was formed, which rules out the occurrence of a racemic background reaction. On the other hand, *Me*HNL, like all HNLs, not only catalyzes the formation of one enantiomer, but also its degradation to the prochiral carbonyl compound.^30^, ^44^, ^47^ Therefore, a decrease in *ee* over time can be ascribed to the ability of the enzyme to speed up the racemization process in this manner. This effect has also been observed in toluene (Figures 1 and 5).

**Figure 2:**
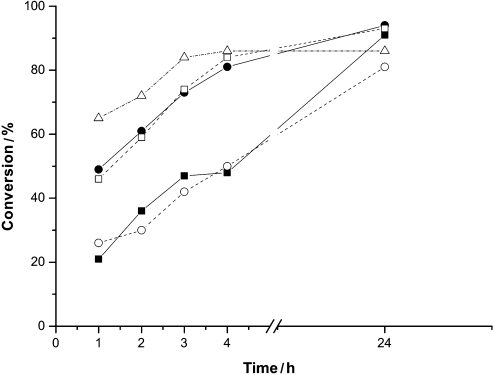
Conversion of **1** in the *Me*HNL-CLEA-catalyzed synthesis of (*S*)-mandelonitrile (**3**) in MTBE with different water concentrations (▪ dry; ○ sat.; • 0–2; □ 2–7; ▵ 7–12).

**Figure 3:**
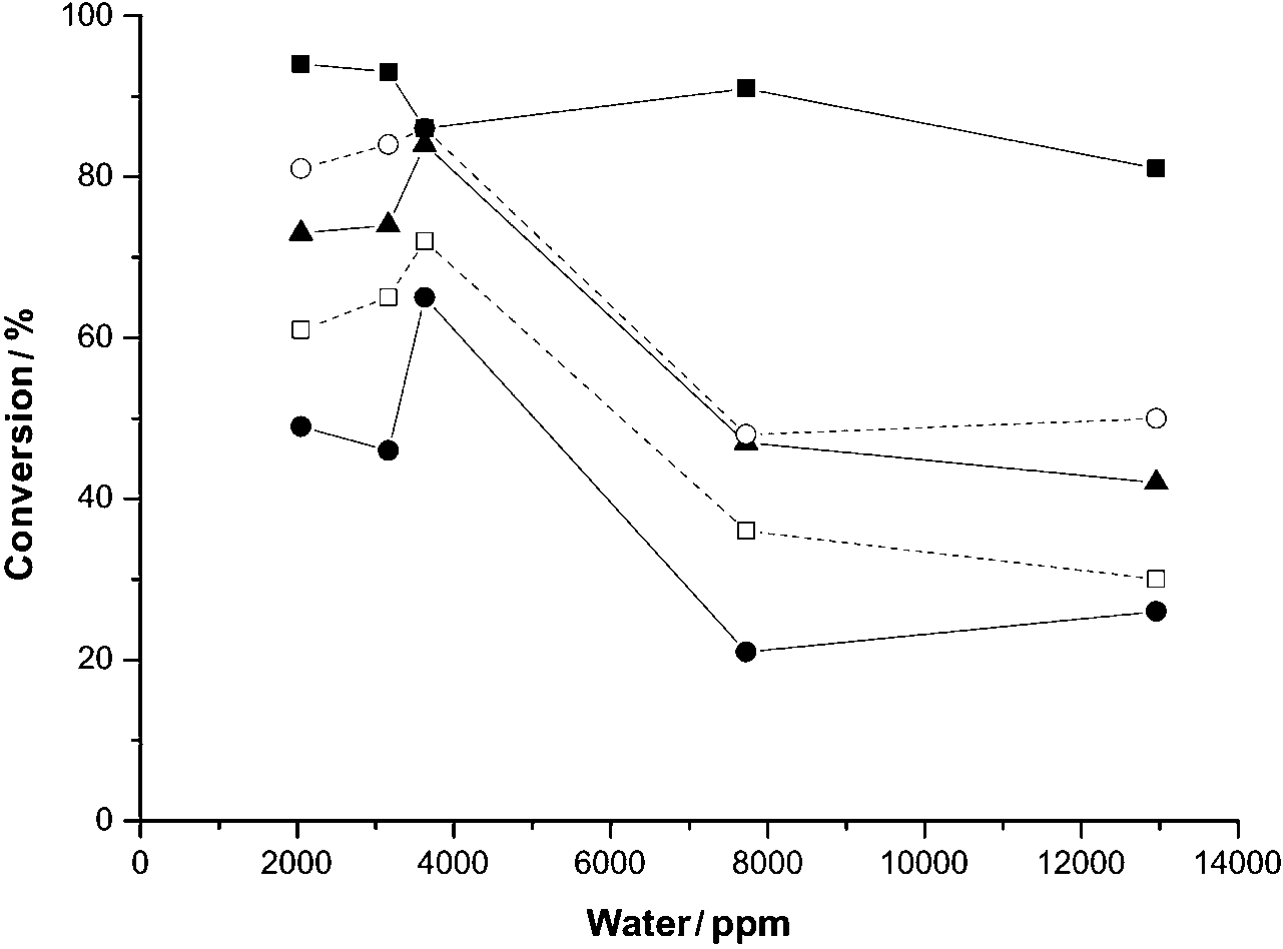
Effect of water concentration on the conversion of **1** in the *Me*HNL-CLEA-catalyzed synthesis of (*S*)-mandelonitrile (**3**) in MTBE (• 1 h; □ 2 h; ▴ 3 h; ○ 4 h; ▪ 24 h)

The influence of water concentration on the conversion in MTBE is significant (Figures 2 and 3): at low water concentration, insufficient hydration of the enzyme lowers the activity. This is in line with results described by Costes et al.^8^ On the other hand, when the water concentration is increased above the optimum level, the activity is reduced once more. This might be due to a high concentration of water in the active site that needs to be displaced by cyanide for the reaction to take place.^66^ In all cases, the reaction was found to be faster when the salt pairs were employed to adjust the *a*_W_ before the reaction. Systems with higher initial water concentrations (water-saturated and “dry” MTBE) displayed remarkably lower initial rates. After 24 h, all of the systems had reached the conversion equilibrium value, and no further reaction was observed.

**Figure 4:**
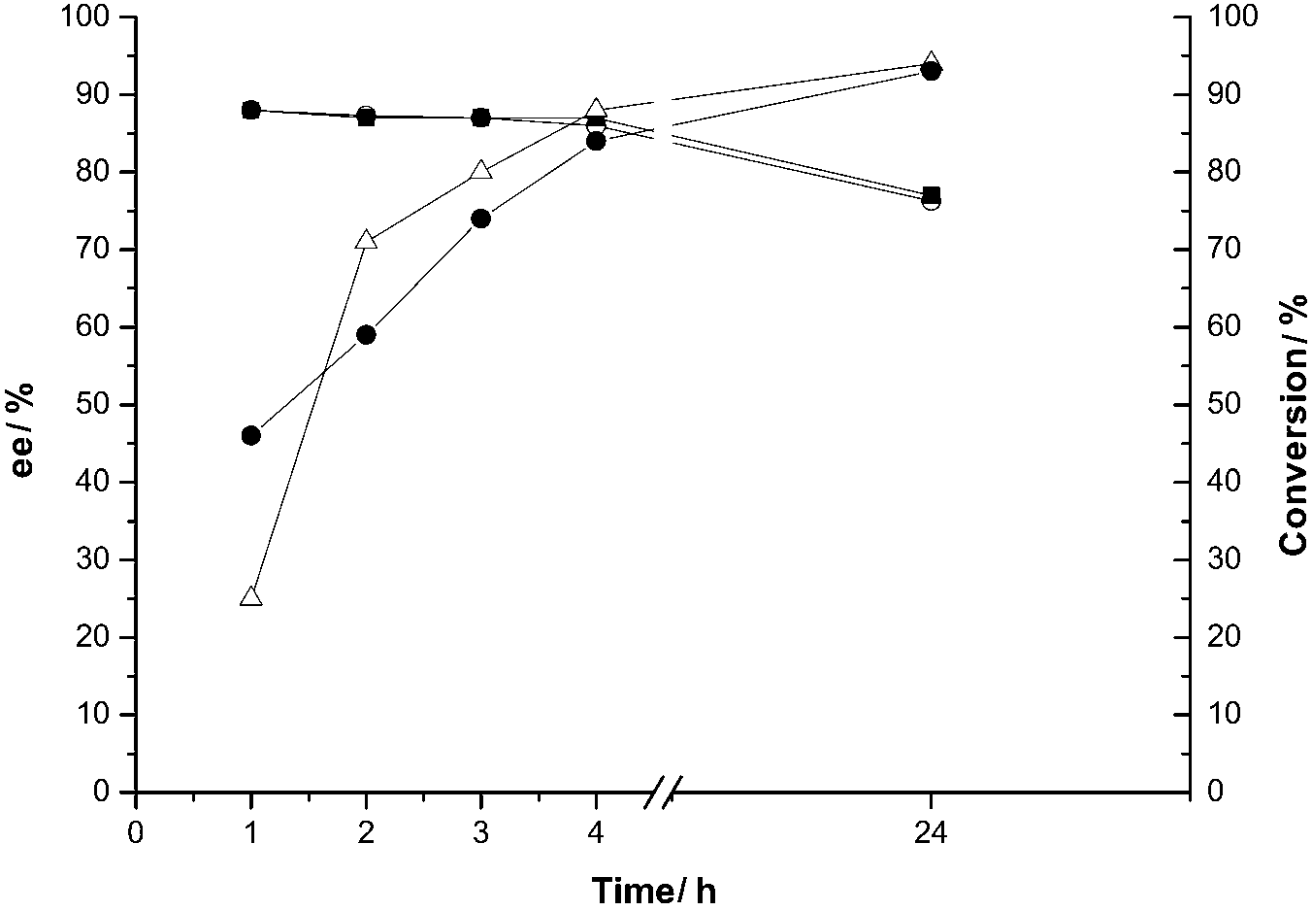
Conversion of **1** and *ee* of (*S*)-mandelonitrile (**3**) in the *Me*HNL-CLEA-catalyzed hydrocyanation in MTBE with and without the salt pair Na_2_HPO_4_⋅2 H_2_O/Na_2_HPO_4_⋅7 H_2_O in the reaction medium (with salt: ▵=conv., ○=*ee*; without salt: •=conv., ▪=*ee*).

The final water concentration in the MTBE highlights the ability of this solvent to take up water trapped in *Me*HNL-CLEA in the course of the reaction (Table 1). To clarify this, the reaction was also performed in parallel both under the usual conditions (salts removed just before adding all of the reagents) and by keeping the pair (Na_2_HPO_4_⋅2 H_2_O/Na_2_HPO_4_⋅7 H_2_O) in the reaction flask using a tea-bag approach. Remarkably, the water concentration increased even more, by about one order of magnitude over 24 h, when the salt pair was allowed to remain in the reaction mixture (Table 2). This unexpected effect resulted only in a minor change of rate, and did not affect the *ee* (Figure 4). However, when *Me*HNL-CLEA was suspended in MTBE and stirred for 24 h in the presence of the same salt pair, but without reagents (Table 2, control experiment no. 1), the water concentration was found to remain almost constant. Thus, the increase in water concentration is a feature purely of the reagents (Table 2, control experiment no. 2). The latter significantly increase the polarity of the solvent; as a consequence, the release of water by the enzyme and the salt pair is enhanced, and the water concentration corresponding to the given *a*_W_ value (which remains constant) is increased accordingly.

**Table 2:** Initial and final water concentrations for (*S*)-mandelonitrile (**3**) synthesis in MTBE in the presence and absence of Na_2_HPO_4_**⋅**2 H_2_O/Na_2_HPO_4_**⋅**7 H_2_O.

	Salt kept during the reaction	Salt removed (standard conditions)	Control experiments	
			1	2
initial water concentration [ppm]	3500[a]	3200[a]	4200[c]	3100[d]
final water concentration [ppm]	32 000[b]	9600[b]	5500[c]	10 100[d]

[a] Measured after stirring the CLEA in the solvent for 1 h in the presence of the salt pair “2–7”.

[b] Measured for the reaction mixture after 24 h.

[c] The CLEA was stirred in the solvent in the presence of the salt pair “2-7” but without reagents for 24 h. The water concentration was measured after 1 h and after 24 h.

[d] The reagents were stirred in the solvent in the presence of the salt pair “2-7” but without the CLEA. The water concentration was measured after 1 h and after 24 h.

**Figure 5:**
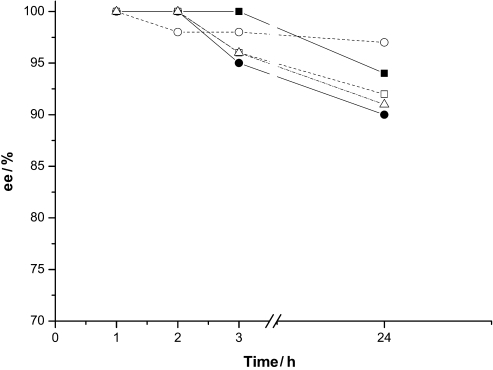
Variation in *ee* of (*S*)-mandelonitrile (**3**) in the *Me*HNL-CLEA-catalyzed hydrocyanation in toluene at different water concentrations (▪ dry; ○ sat.; • 0–2; □ 2–7; ▵ 7–12).

**Toluene as solvent**: The toluene-based systems (Table 3) contained approximately one or even two orders of magnitude less water than those based on MTBE (Table 1), even though the *a*_W_ was the same when using salt pairs. In the course of the reaction, the water concentration in each system increased noticeably due to the continuous release of water from *Me*HNL-CLEA into the solvent. Hydrocyanations performed in a less polar solvent such as toluene gave better *ee* values than those performed in MTBE, and showed a less pronounced influence of the water concentration on the enantioselectivity (Figure 5). As in MTBE, however, the highest enantioselectivities were observed in the systems with the highest water concentrations, that is, “dry” and water-saturated toluene (Table 3). The three systems containing a salt pair showed very similar behaviour and afforded slightly lower *ee* values.

**Table 3:** Initial and final water concentrations for reactions in toluene.

	“Dry”	Sat.	0–2	2–7	7–12
water concentration of solvent [ppm]	250[a]	470[b]	–	–	–
water concentration of solvent + salt pair [ppm]	–	–	300[c]	370[c]	400[c]
initial water concentration of the reaction mixture [ppm]	460[d]	580[d]	40[e]	70[e]	180[e]
final water concentration of the reaction mixture [ppm][f]	8300	10 600	10 000	4400	5600

[a] Measured for a sample of anhydrous solvent from Sigma–Aldrich.

[b] Measured for a sample of water-saturated solvent.

[c] In a separate experiment, the salt pairs were suspended in the anhydrous solvent and the system was stirred for 1 h before measuring the water concentration.

[d] Measured after stirring the CLEA in the solvent for 1 h.

[e] Measured after stirring the CLEA and the salt pair in the solvent for 1 h.

[f] Measured for the reaction mixture after 24 h.

**Figure 6:**
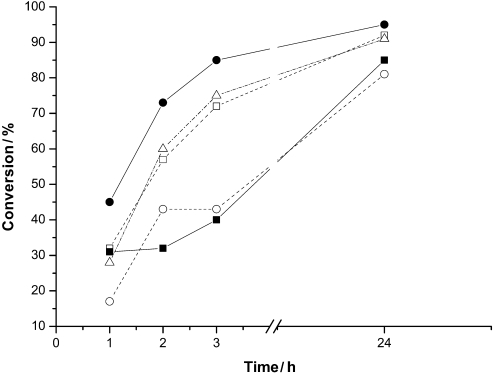
Conversion of **1** in the *Me*HNL-CLEA-catalyzed synthesis of (*S*)-mandelonitrile (**3**) in toluene with different water concentrations (▪ dry; ○ sat.; • 0–2; □ 2–7; ▵ 7–12).

**Figure 7:**
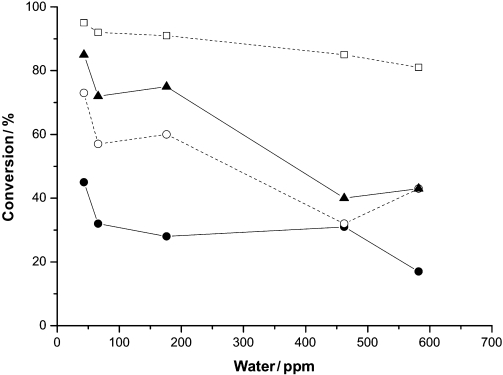
Effect of water concentration on the conversion of **1** in the *Me*HNL-CLEA-catalyzed synthesis of (*S*)-mandelonitrile (**3**) in toluene. (• 1 h; ○ 2 h; ▴ 3 h; □ 24 h)

Considering the conversions, they were slightly lower than those in MTBE, even though an inverse correlation between enzymatic activity and solvent hydrophilicity has been described for other enzymes.^62^, ^67^ As in MTBE, a positive effect of low water concentrations on the reaction rate was observed (Figures 6 and 7).

**Figure 8:**
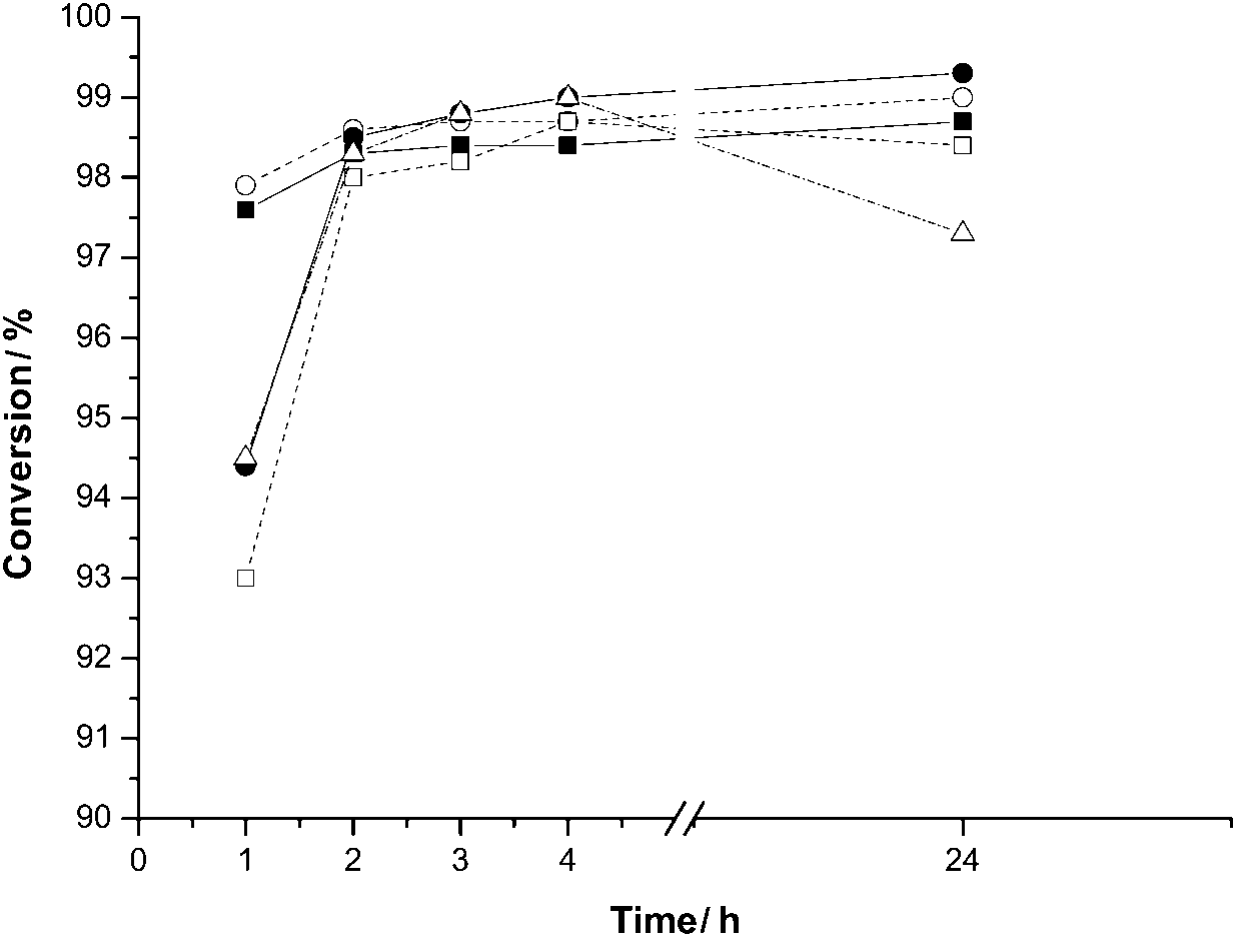
Conversion of **1** in the *Me*HNL-CLEA-catalyzed synthesis of (*S*)-mandelonitrile (**3**) in octane at different water concentrations (▪ dry; ○ sat.; • 0–2; □ 2–7; ▵ 7–12).

**Octane as solvent**: Choosing the hydrocarbon octane (Table 4) allowed us to explore the effect of a very hydrophobic solvent, with log*P*=5.15. Medium hydrophobicity is known to affect enzyme activity in hydrocyanations mediated by various supported HNLs;^8^ indeed, it was shown that increasing log*P* in *Hb*HNL-mediated hydrocyanation resulted in a dramatic increase in enzyme activity. More generally, hydrophobic solvents enhance enzyme activity with respect to their hydrophilic counterparts because they are less effective in stripping the essential enzyme-bound water.^67^ Similar effects were expected to be manifested in our study. However, replacing MTBE (log*P*=0.94) with the less polar toluene (log*P*=2.73) did not improve the reaction rate significantly; rather, it did the contrary. For octane, however, the effect was dramatic (Figure 8): almost full conversion was reached after 1 h. In addition to the effect of the high log*P* on the enzyme, this can be explained in terms of the poor solubility of mandelonitrile and acetone cyanohydrin in octane, while benzaldehyde dissolves completely. Thus, the formed mandelonitrile tends to separate from the reaction mixture. This in situ product removal shifts the equilibrium dramatically (Figure 8). On the other hand, the partitioning of acetone cyanohydrin between the aqueous and organic phases depends on log*P*; the concentration of this reagent in the water layer around the enzyme becomes much higher in octane than in toluene or MTBE, thus inducing higher enzymatic activity.^8^

**Table 4:** Water concentrations and final (*S*)-mandelonitrile *ee* for reactions in octane.

	“Dry”	Sat.	0–2	2–7	7–12
water concentration of solvent [ppm]	40[a]	70[b]	–	–	–
water concentration of solvent + salt pair [ppm]	–	–	50[c]	50[c]	60[c]
initial water concentration of the reaction mixture [ppm]	50[d]	70[d]	10[e]	20[e]	50[e]
final water concentration of the reaction mixture [ppm][f]	50	80	50	40	60
final *ee* [%] of (*S*)-**3**	86	88	86	87	89

[a] Measured for a sample of anhydrous solvent from Sigma–Aldrich.

[b] Measured for a sample of water-saturated solvent.

[c] In a separate experiment, the salt pairs were suspended in the anhydrous solvent and the system was stirred for 1 h before measuring the water concentration.

[d] Measured after stirring the CLEA in the solvent for 1 h.

[e] Measured after stirring the CLEA and the salt pair in the solvent for 1 h.

[f] Measured for the reaction mixture after 24 h.

Final *ee* values for mandelonitrile were determined upon addition of isopropanol to the reaction mixture after 24 h. These were similar to those observed in MTBE and lower than those in toluene (Table 4; Figures 1 and 5). It is known that enzyme enantioselectivity in nonaqueous media can depend markedly on the solvent,^68^–^71^ and in some cases even complete inversion of enantioselectivity^2^ following a change of solvent has been reported. *Me*HNL-CLEA displayed (*S*)-selectivity in all of the media screened, and only a small decrease in the *ee* of (*S*)-mandelonitrile was observed when using octane or MTBE. Such differences compared to toluene can be attributed to the influence of the solvent on the enzyme conformation.

**Figure 9:**
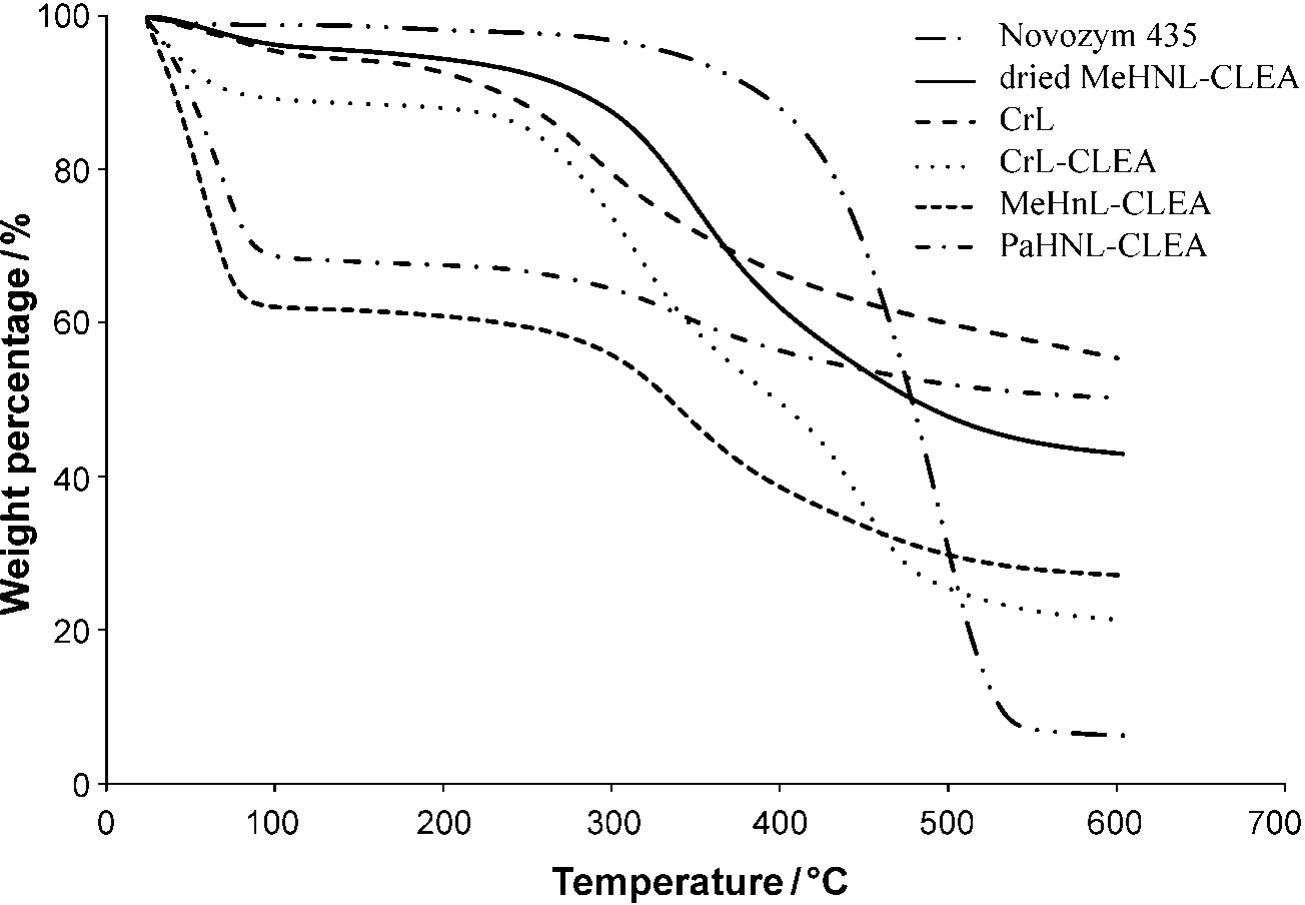
TGA of different enzyme preparations in nitrogen at a heating rate of 10 °C min^−1^.

An observation made for all of the solvents was a significant increase in the water concentration in the reaction mixture after 24 h. This was particularly prominent for MTBE and toluene. The water concentrations of all reaction components were then measured (Table 5), and were found to be considerable. The water trapped in commercially available *Me*HNL-CLEA was determined by thermogravimetric analysis (TGA) to be 38 % by weight (Figure 9). The extent to which this water is released into the reaction medium varies greatly depending on the solvent; toluene and especially MTBE showed a marked ability to take up the water from the immobilized enzyme. The release of water from the enzyme most probably depends on the catalytic activity of the enzyme and the polarity changes of the solvent due to the presence of the reagents. It has been shown^72^, ^73^ that the binding of transition-state analogues strengthens the interactions between subunits and between protein groups and catalytic site ligands. Thus, when benzaldehyde binds to the active site of *Me*HNL, and hydrocyanation takes place, a significant conformational change occurs. The enzyme adopts a compact and rigid conformation, thereby reducing its flexibility. As a consequence, internal water molecules are released into the solvent, accounting for the final water concentration.^73^ These results shed new light on the drastic loss of activity observed for immobilized *Hb*HNL after incubation in organic solvent saturated with buffer in the presence of phenylpropanal.^74^ Binding of this substrate resulted in a tight complex, which rapidly released the internal water. As a consequence, the activity dropped. In the course of such a reaction, the reagents, intermediates (HCN), and products cause the solvent polarity to change. With an increase in polarity, the water trapped in the CLEA will escape into the reaction mixture. The relationship between the residual water trapped in commercial *Me*HNL-CLEA and its catalytic activity was highlighted by an additional experiment. By dehydration using P_2_O_5_, the internal water in this CLEA was reduced to 4 % (as determined by TGA; Figure 9). The dehydrated enzyme showed a dramatic drop in both activity and enantioselectivity in the hydrocyanation of benzaldehyde in either dry MTBE or toluene according to the described protocol. However, the catalytic activity was reversibly restored upon rehydration (decomposition of mandelonitrile in aqueous buffer).^75^ This result is analogous to the reactivation upon rehydration described by Sym^76^ for porcine pancreas lipase. It is thus clear that the internal water is essential for the activity of *Me*HNL-CLEA, as has previously been described for other enzymes.^77^ The deleterious effect of dehydration on the catalytic activity of enzymes is a common feature of different proteins,^67^, ^78^, ^79^ and is often a consequence of significant denaturation.

**Table 5:** Water concentrations of each of the reaction components.

System	Water concentration [ppm]
MTBE[a]	200
*Me*HNL-CLEA[b]	7800
benzaldehyde (**1**)[c]	400
acetone cyanohydrin (**2**)[c]	5100
total	13 500

[a] Directly from a Sure/Seal bottle.

[b] The CLEA was suspended in dry MTBE and stirred for 24 h. A sample of solvent was then injected into the Karl Fischer titrator.

[c] Samples of freshly distilled reagents were injected directly into the Karl-Fischer titrator.

The water concentrations of several free or immobilized commercial enzymes were also measured by TGA and compared with that of *Me*HNL-CLEA (Figure 9). The latter contains a remarkably higher amount of water than *Candida rugosa* lipase (both free and immobilized as CLEA) or commercial Novozym 435, but the amount is comparable to that held by the CLEA of the (*R*)-selective and structurally unrelated HNL from *Prunus amygdalus*.^31^

## Conclusion

The *Me*HNL-CLEA-catalyzed hydrocyanation of benzaldehyde has proved to be an excellent model reaction for investigating the role of residual water in enzymatic transformations in organic solvents. The immobilized enzyme was stable in all of the screened organic solvents and a slight positive effect of increasing log*P* on the product *ee* was observed. Remarkably higher conversions achieved in octane may be partly attributed to separation of the product during the reaction, which shifts the reaction equilibrium.

It has clearly been demonstrated that the concentration of water in an organic medium should not be neglected, as it can affect both activity and selectivity. In the case of *Me*HNL-CLEA, an increase in the water concentration for a given solvent induced higher enzyme enantioselectivity. A direct correlation between enzyme flexibility and enantioselectivity can be inferred. This conclusion is at variance with that drawn by Rariy and Klibanov^57^ for subtilisin Carlsberg and α-chymotrypsin.

In general, higher activity was observed at relatively low water levels. However, thorough drying of the enzyme prior to the reaction did lead to reversible deactivation. The amount of this residual water in commercial *Me*HNL-CLEA was found to be as high as 38 % (w/w). Its role is essential in ensuring enzymatic activity when performing hydrocyanations in dry organic solvents. The release of enzyme-bound water into the solvent accounts for the observed increase of water concentration during the reaction.

For a better understanding of enzymatic reactions in organic solvents, the water contents of enzyme preparations and reagents should always be taken into account. Furthermore, deeper insight into the complex role of water can be obtained by measuring the final water concentration and comparing it with the initial one.

## Experimental Section

CAUTION: All procedures involving HCN were performed in a well-ventilated fume-hood equipped with an HCN detector. HCN-containing wastes were neutralized using commercial bleach and stored independently over a large excess of bleach for disposal.

**Enzymes**: *Me*HNL-CLEA, *Pa*HNL-CLEA, and *Candida rugosa* lipase (type VII)-CLEA were supplied by CLEA Technologies B.V. *Candida rugosa* lipase (type VII) was purchased from Sigma Aldrich. Novozym 435 was donated by Novozymes.

**Enzyme activity measurement**: The enzymatic activity of *Me*HNL-CLEA was measured according to reported literature procedures^75^ and was found to be 0.44 U mg^−1^. Samples were prepared by suspending CLEA (32 mg) in pH 6.5 phosphate buffer (3 mL).

**Chemicals**: (±)-Mandelonitrile (Acros Organics, technical grade) was purified through column chromatography (PE/EtOAc 9:1 → 3:7) prior to use. Acetone cyanohydrin was distilled in the presence of 2 % phosphoric acid prior to use and was stored under nitrogen at 4 °C. Benzaldehyde, of analytical grade, was always distilled prior to use and was stored under nitrogen at 4 °C. Anhydrous MTBE (99.8 %), toluene (99.8 %), and octane (>99 %) were purchased from Sigma–Aldrich.

**Analytical methods**: The course of each enzyme reaction was followed by chiral HPLC analysis at 40 °C using a Waters system (Waters 486 UV detector, Waters 515 pump, and Waters 717+ injector) equipped with a Chiralcel OB-H column from Daicel (4.5 μm × 250 mm) and using *n*-heptane/2-propanol (95:5) as solvent (flow rate: 1 mL min^−1^). Retention times: 3.68 min (triisopropylbenzene), 7.00 min (benzaldehyde), 15.81 min ((*R*)-mandelonitrile), 16.83 min ((*S*)-mandelonitrile). Water concentrations in solvents and reaction mixtures were determined by Karl Fischer titration using a Metrohm 831 KF coulometer equipped with a generator electrode with diaphragm, according to the manual provided (determination range: 10 μg–200 mg H_2_O; resolution: 0.1 μg H_2_O; reproducibility: ±3 μg in the range 10–1000 μg H_2_O, 0.3 % or better for values above 1000 μg). All measurements were performed in duplicate and the numbers in the tables are mean values. Water trapped in *Me*HNL-CLEA was measured by thermogravimetry using a Perkin–Elmer TGA7 thermogravimetric analyzer. The measurements were performed under nitrogen atmosphere in the range 25–625 °C at a heating rate of 10 °C min^−1^. The initial sample mass was always in the range 4–12 mg.

**Blank experiment**: Benzaldehyde (100 μL, 1 mmol) was placed first under vacuum (oil pump) and subsequently under nitrogen, and finally dissolved in dry MTBE (1 mL). The nitrogen line was then closed in order to prevent HCN leakage. Triisopropylbenzene (84 μmol, 20 μL) was added and an HPLC sample to determine the initial conditions was prepared by taking 10 μL of reaction mixture, diluting it with *n*-heptane/2-propanol (95:5), and filtering before injection. After 20 min, the initial water concentration was also measured. The reaction was initiated by the addition of a 1.05 m solution of HCN in diisopropyl ether (6 mL, 6 equiv) and was monitored by chiral HPLC over one day. Samples (10 μL) were withdrawn at regular intervals (1, 2, 3, 4, 24 h) according to the described procedure. The final water concentration was determined after 24 h.

**General procedure for the enzymatic hydrocyanation in dry and water-saturated solvents**: *Me*HNL-CLEA (0.44 U mg^−1^, 15 U) was placed first under vacuum (oil pump) and subsequently under nitrogen, and finally suspended in solvent (1.7 mL). The nitrogen line was then closed in order to prevent HCN leakage. A sample (10 μL) was taken after 1 h by means of a syringe to measure the initial water concentration. Benzaldehyde (1.0 mmol, 100 μL) and the internal standard triisopropylbenzene (84 μmol, 20 μL) were added and after a few minutes an HPLC sample to determine the initial conditions was prepared by taking 10 μL of the reaction mixture, diluting it with *n*-heptane/2-propanol (95:5), and filtering before injection. The reaction was initiated by the addition of acetone cyanohydrin (560 μL, 6 equiv) and monitored by chiral HPLC over one day. Samples (10 μL) were withdrawn at regular intervals (1, 2, 3, 4, 24 h) according to the described procedure. The final water concentration was determined after 24 h.

**General procedure for the enzymatic hydrocyanation in the presence of a salt pair**: The salt pairs (0.5 g of each salt) were weighed onto a filter paper (Rotilabo, Ø 55 mm), which was folded and then added to a flask already containing *Me*HNL-CLEA (0.44 U mg^−1^, 15 U). The flask was placed under vacuum and then nitrogen was admitted. The solvent (MTBE, toluene, or octane; each stored under nitrogen) was then added. The nitrogen line was closed and the system was stirred at room temperature for 1 h; a sample was then taken by means of a syringe to determine the initial water concentration by Karl Fischer titration. The salts were then removed. Benzaldehyde (1.0 mmol, 100 μL) and triisopropylbenzene (84 μmol, 20 μL) were added and, after a few minutes, a HPLC sample to determine the initial conditions was prepared by taking 10 μL of the reaction mixture, diluting it with *n*-heptane/2-propanol (95:5), and filtering before injection. The reaction was initiated by the addition of acetone cyanohydrin (560 μL, 6 equiv) and monitored by chiral HPLC over one day. Samples (10 μL) were withdrawn at regular intervals (1, 2, 3, 4, 24 h) according to the described procedure. The final water concentration was determined after 24 h.
